# Plant Stage, Not Drought Stress, Determines the Effect of Cultivars on Bacterial Community Diversity in the Rhizosphere of Broomcorn Millet (*Panicum miliaceum* L.)

**DOI:** 10.3389/fmicb.2019.00828

**Published:** 2019-04-24

**Authors:** Xiaofan Na, Xiaoning Cao, Caixia Ma, Shaolan Ma, Pengxin Xu, Sichen Liu, Junjie Wang, Haigang Wang, Ling Chen, Zhijun Qiao

**Affiliations:** ^1^School of Life Sciences, Ningxia University, Yinchuan, China; ^2^Key Laboratory of Crop Gene Resources and Germplasm Enhancement on Loess Plateau, Ministry of Agriculture, Taiyuan, China; ^3^Shanxi Key Laboratory of Genetic Resources and Genetic Improvement of Minor Crops, Institute of Crop Germplasm Resources of Shanxi Academy of Agricultural Sciences, Taiyuan, China

**Keywords:** broomcorn millet, cultivar, bacterial community, rhizosphere, drought stress, plant age

## Abstract

Broomcorn millet (*Panicum miliaceum* L.) is one of the oldest domesticated crops and has been grown in arid and semiarid areas in China since 10,000 cal. BP. However, limited information is available about how bacterial communities within the rhizosphere of different broomcorn millet cultivars respond to drought stress. Here, we characterized the changes in the rhizobacterial assemblages of two broomcorn millet cultivars, namely, *P. miliaceum* cv. *HeQu Red* (HQR) and *P. miliaceum YanLi 10* (YL10), from the jointing stage to the grain filling stage after they were exposed to a short-term drought stress treatment at the seedling stage. Drought significantly inhibited the growth of both cultivars, but the effect on YL10 was higher than that on HQR, indicating that the drought tolerance of HQR was greater than that of YL10. *Proteobacteria* (33.8%), *Actinobacteria* (21.0%), *Acidobacteria* (10.7%), *Bacteroidetes* (8.2%), *Chloroflexi* (6.3%), *Gemmatimonadetes* (5.9%), *Firmicutes* (3.5%), *Verrucomicrobia* (2.9%), and *Planctomycetes* (2.7%) were the core bacterial components of broomcorn millet rhizosphere as suggested by 16S rDNA sequencing results. The diversity and composition of bacterial rhizosphere communities substantially varied at different developmental stages of broomcorn millet. As the plants matured, the richness and evenness of the rhizobacterial community significantly decreased. Principal coordinate analysis showed that the structure of the bacterial rhizosphere community changed notably only at the flowering stage between the two cultivars, suggesting a stage-dependent effect. Although drought stress had no significant effect on the diversity and structure of the bacterial rhizosphere community between the two cultivars, differential responses to drought was found in Actinobacteria and *Acinetobacter*, *Lysobacter*, *Streptomyces*, and *Cellvibrio*. The relative abundance of Actinobacteria and *Lysobacter*, *Streptomyces*, and *Cellvibrio* in the YL10 rhizosphere was stimulated by the drought treatment compared with that in the HQR rhizosphere, whereas the opposite effect was found in *Acinetobacter*. Our results suggested that the effects of cultivars on bacterial rhizosphere communities were highly dependent on plant developmental stage, reflecting the genetic variations in the two broomcorn millet cultivars.

## Introduction

The rhizosphere is a specific microenvironment in terrestrial ecosystems, and it permits a sophisticated exchange between numerous organisms and their environment. Diverse bacteria, fungi, and viruses live in the rhizosphere of plants, and many of these microorganisms facilitate various processes, including nutrient uptake, hormone production, and disease and pest prevention (de Vrieze, 2015). Given the vital roles of these organisms to plant growth and health, certain microbes, such as nitrogen-fixing bacteria and arbuscular mycorrhizal fungi, have been found to help plants successfully live under stressful conditions (Sánchez-Blanco et al., 2004) and can be used to increase crop yield in agro-ecosystems with minimal use of chemical fertilizers and pesticides (Glick, 2014). Therefore, understanding the mechanisms of plant–microorganism interactions within the rhizosphere can help us tackle challenges associated with the sustainability and productivity of agro-ecosystems.

For annual and perennial plant species, plant stage is an important factor that drives shifts in microbial rhizosphere community composition under field and greenhouse conditions (Houlden et al., 2008; Micallef et al., 2009; Na et al., 2017a). As plants develop, variations in the physiological status of plants affect the growth of distinct microbial groups within the rhizosphere mediated by variation in the composition of plant root exudates and depositions (Mougel et al., 2006). The diversity and quantity of carbon flow to and from the roots into the rhizosphere change with plant development (Swinnen et al., 1994; Duineveld et al., 2001). These results have suggested that plants can initiate and actively recruit their rhizosphere microbiome to meet their requirements at a given developmental stage. Peiffer et al. (2013) found a small but significant proportion of heritable variation in bacterial rhizosphere diversity among 27 modern maize inbreds under field conditions. Similar results have also been found in the rhizosphere of different genotypes of *Arabidopsis thaliana* under controlled conditions (Bulgarelli et al., 2012; Lundberg et al., 2012). Given that the genotype of a plant corresponds to its phenotype, the different responses of rhizosphere microbiota may reflect the differences in physiological conditions among genotypes or cultivars at a specific developmental stage. Thus, analyzing interactions between microbiota and their host plants may be an alternative way to identify plant alleles that control plant performance (Peiffer et al., 2013).

Shifts in plant–bacterium interactions in the rhizosphere have been observed when plants are subjected to drought stress, and research on how this interaction contributes to plant drought tolerance has been widely performed (Dimkpa et al., 2009; Xu et al., 2018). As a response to water deficit, osmotolerant rhizobacteria increase the synthesis of osmolytes, such as glycine betaine, and enhance the drought tolerance of host plants (Dimkpa et al., 2009). These osmotolerant bacteria can also stimulate root growth and reconstruct the root architecture of host plants by producing indole-3-acetic-acid (Yuwono et al., 2005). Improved root growth enhances the efficiency of water acquisition and nutrient uptake of host plants under drought stress. However, most studies on the plant–rhizobacterium interactions under drought stress have focused on various species, such as rice (Yuwono et al., 2005), maize (Casanovas et al., 2002), tomato (Mayak et al., 2004), and common bean (German et al., 2000), which do not have notable drought resistance. The response of the bacterial rhizosphere community of plant species with inherently low water requirements to water deficit has yet to be further investigated.

Broomcorn millet (*P. miliaceum* L.), also known as common, hog or proso millet, is one of the oldest cultivated and domesticated crops. In 10,000 cal. BP, this crop was cultivated in China (Hunt et al., 2008, 2014); since then, it has been considered a staple cereal in Northern China (Hunt et al., 2011). It has several unique characteristics among cereal species in terms of its ecology, geography, and cultivation history (Hunt et al., 2011). Among cereal crops, broomcorn millet has the lowest water requirement and shortest growth period (Baltensperger, 2002). It also has a low nutrient requirement (Rajput et al., 2016) and can be cultivated in marginal agricultural lands, where the cultivation of other cereals dose not succeed, because it can tolerate drought and high-temperature stresses and is well adapted to saline–alkaline soils (Hunt et al., 2011; Wang et al., 2016). Currently, this important cereal is cultivated in large areas in arid and semi-arid steppe regions in Northern China, Russia, Central Asia, and North America (Zohary and Hopf, 2000).

Although previous studies investigated the agronomic trait diversity (Wang et al., 2016), genetic diversity (Hunt et al., 2011; Liu et al., 2016; Rajput et al., 2016), and stress tolerance mechanisms (Karyudi and Fletcher, 2002; Dai et al., 2011; Zhang et al., 2012) of broomcorn millet, a comprehensive understanding of its diversity and the structure of its rhizosphere bacterial assemblages is lacking. Plant–microbe interactions in the rhizosphere are genetically controlled (Peiffer et al., 2013). Hence, broomcorn millet provides an ideal system to reveal the mechanisms of heritable plant–microorganism interactions and the role of bacterial rhizosphere populations in drought tolerance. For these purposes, we characterized the rhizosphere bacterial community across two cultivars of broomcorn millet under drought stress by monitoring their different developmental stages via pyrosequencing 16S rRNA gene amplicons. On the basis of previous studies suggesting that bacterial rhizosphere community diversity was driven by the developmental stage of host plants and our preliminary experiments demonstrating that the two broomcorn millet cultivars differed in drought tolerance, we hypothesized that (1) the effect of cultivar on the bacterial community composition depended on the developmental stage of the plant; (2) drought profoundly affected rhizobacterial community structure; and (3) the responsive pattern of the relative abundance of individual bacterial populations to drought depended on the cultivar of broomcorn millet. Knowledge about the temporal shift of plant-associated microbial communities would help enhance our understanding of the differences in the drought tolerance of broomcorn millet cultivars and the interactions between this oldest drought-tolerant crop and its associated microbiota.

## Materials and Methods

### Experimental Design

Pot experiments, which had a completely randomized block design with three replicates, were conducted with two broomcorn millet cultivars, namely *P. miliaceum* L. cv. *HeQu Red* (HQR) and *YanLi 10* (YL10), at the Hequ Research Station of the Shanxi Academy of Agricultural Sciences (39°08′20.78″N, 110°14′18.74″E) at the eastern fringe of the Loess Plateau, Shanxi Province, China. The soil samples (sandy loam) were collected from a local agricultural field (soil auger, 5 cm in diameter and 20 cm in length), which had not been used previously to grow broomcorn millet. The field soils were air dried and sieved to 2 mm to remove rocks and plant debris.

Broomcorn millet plants were planted in pots (height; 35 cm; diameter: 30 cm) containing 10 kg of the sieved dry soil. All of the experiments were conducted in a greenhouse to avoid rainfall. Thirty surface-sterilized seeds bleached for 5 min and rinsed at least three times with sterile water were planted in each pot on June 11, 2015, and 16 seedlings were kept after germination. After 55 days of growth, the soil water content of half of the pots decreased to 15% water holding capacity without watering (about 4 days) and maintained at 15% for another 15 days by weighing the pots every day (drought stress), whereas the remaining pots were maintained at 55% of water holding capacity (control). The experiment consisted of 36 samples (3 replicates × 2 cultivars × 2 treatments × 3 stages). Two pots were randomly selected from each of the replicates, and all of the plant roots were bulked as a single sample. Therefore, 72 pots were used in this study. The experimental procedure is shown in detail in Supplementary Figure S1.

### Sampling

The rhizosphere soil was first sampled after 15 days of drought stress (i.e., the jointing stage). The water holding capacity of the remainder pots was returned to 55%. Additional samples were harvested 10 (flowering stage) and 20 (grain filling stage) days after the first 15 days in the control and drought stress treatments. Destructive sampling was performed 15, 25, and 35 days after the drought treatment, and all of the plants (total plants = 32) in two random pots were pooled and considered as one replicate (total replicates = 3). After the loosely adhered soil was removed by shaking, the root samples of the harvest plants were transferred into a 5 ml sterilized tube and stored in ice immediately. The roots with adhered soil were washed with 5 ml of 0.9% sterile NaCl solution to collect the rhizosphere soil. The resulting solution was centrifuged at 12,000 rpm and at 4°C for 10 min, and deposition was defined as the rhizosphere soil sample. These rhizosphere soils were then stored at –20°C until further analysis.

After the last sampling was conducted, the shoots of all of the plants were harvested on September 30, 2015, and used for quantifying the agronomic traits of plant height, number of internodes, culm diameter, and panicle length. Plants were dried to constant weight at 60°C before the dry weights of panicles and grains were determined.

### Total Genomic DNA Extraction and Amplicon Generation

Total genomic DNA was extracted from the rhizosphere soil samples by using a PowerSoil DNA isolation kit (MoBio, United States) in according with the manufacturer’s instructions. DNA integrity and purity were monitored on 1% agarose gels. Partial 16S rDNA-based high-throughput sequencing was performed to detect the bacterial diversity and composition of each sample according to Caporaso et al. (2010). The V4 region of the 16S rRNA gene was amplified with the specific primer 515F and 806R with a 6 bp error-correcting barcode unique to each sample. PCR was conducted in 30 μl reactions with 15 μl of Phusion High-Fidelity PCR Master Mix (New England Biolabs, United Kingdom), approximately 10 ng of template DNA, and 0.2 μM forward and reverse primers. Thermal cycling involved initial denaturation at 98°C for 1 min, followed by 30 cycles of denaturation at 98°C for 10 s, annealing at 50°C for 30 s, elongation at 72°C for 60 s, and a final round of elongation at 72°C for 5 min. The PCR products were detected on 2% agarose gel, and the samples with bright main strips between 200 and 300 bp were chosen and purified for further analyses.

### Library Preparation and Sequencing

Amplicon libraries were generated using a NEB Next Ultra^TM^ DNA Library Prep kit for Illumina (NEB, United States) in accordance with the manufacturer’s recommendations, and index codes were added. Library quality was assessed using a Qubit 2.0 fluorometer (Thermo Fisher Scientific) and an Agilent Bioanalyzer 2100 system. Subsequently, high-throughput sequencing was performed on an Illumina MiSeq 2500 platform in Novogene (Beijing, China), and 250 bp/300 bp reads were generated. The paired end reads were merged using FLASH (Magoč and Salzberg, 2011) and then assigned to each sample based on the unique barcodes. Sequence analysis was performed by the UPARSE software package by using UPARSE–OTU and UPARSE–OTU ref algorithms. UPARSE pipeline^1^ was utilized to cluster the sequences into operational taxonomic units (OTUs) at a minimum pair-wise identity of 97%. We chose representative sequences for each OTU and used the RDP Classifier (Wang et al., 2007) to annotate taxonomic information for each representative sequence. The sequencing of bacterial 16S rRNA gene amplifications from all of the rhizosphere samples resulted in a total of 641,244 high-quality reads. The results of quality control confirmed that these data could be used for further analysis (Supplementary Table S1). Then, bacterial community alpha diversity indices, such as the Shannon index, OTU number (the number of total OTU observed), and beta diversity based on weighted/unweighted UniFrac distance, were calculated with QIIME (Caporaso et al., 2010). All of the tags in the Core Set (GreenGene data base) were aligned using PyNAST (Version 1.2) to analyze the phylogenetic relationship of the OTUs at the phylum level. The unweighted pair group method with arithmetic mean (UPGMA) clustering was calculated with QIIME.

### Statistical Analysis

One-way ANOVA followed by Tukey’s *post hoc* comparisons was conducted to detect whether the paired data among different treatments were significantly different (*n* = 3). Before analysis was performed, non-normal data were log transformed to achieve Gaussian distribution as checked via a Shapiro–Wilk test. Multi-factor ANOVA was performed by using SPSS version 22.0 (IBM Corp., Armonk, NY, United States) to examine the individual and interactive effects of drought stress and cultivar on the growth of broomcorn millet and the effects of drought stress, cultivar, and developmental stage of broomcorn millet on the α diversity index of rhizosphere bacterial community. Principal coordinate analysis (PCoA) based on the weighted UniFrac distance was performed with vegan package in *R* (v 3.4.3) to test the differences between cultivars, drought stress, and developmental stage on bacterial community composition. Variance partitioning was simultaneously carried out to determine the explanation rates of cultivar, drought, and plant stage on community variance across the samples. Variance partitioning was further conducted on the basis of the data collected only at the stage to detect the explanation rate of cultivar on the variation in bacterial community at the flowering stage. Prior to PCoA and variance partitioning analysis, relative abundance data were Hellinger transformed.

A heat map of the 35 most-abundant bacterial phyla was plotted using *R* to systematically analyze the variation patterns in relative abundance among different developmental stages. The relative abundance of the dominant bacterial rhizosphere phyla (i.e., 10 most dominant) and genera (i.e., 50 most dominant) under drought stress was normalized by dividing the relative abundances of the bacteria under control condition to test whether the individual bacterial rhizosphere population of the two cultivars responded differently to drought stress. Two-factor ANOVA was then performed to examine the individual and interactive effects of the cultivar and developmental stage of broomcorn millet on the variations in the ratio. This multiple testing was an efficient method for screening bacterial populations, which differed in their responses to drought between cultivars, although introducing increased type I errors was possible (Finner and Roters, 2002). Bacterial responsive pattern, which was significantly affected by cultivar, was further calculated using the following equation: (relative abundance under drought/relative abundance under control) -1 and compared using a Tukey *post hoc* test at each developmental stage.

## Results

### Effects of Drought Stress on the Growth of Broomcorn Millet

The phenotype of the two cultivars grown in the control treatment without drought stress significantly differed (Table 1 and Supplementary Table S2). For example, HQR had more internodes, wider diameter culms, and shorter panicles than YL10 (Supplementary Table S2). However, drought stress had a marked effect on plant phenotype (Supplementary Table S2). The drought-exposed plants were shorter than the control plants. The panicle of the former was also shorter than that of the latter. Furthermore, the dry weight of panicles and grains of the former was lower than that of the latter, but the number of internodes and culm diameter of the drought-exposed plants were higher than those of the control plants (Table 1 and Supplementary Table S2). Two-way ANOVA revealed that only the length of panicle was significantly affected by the interactive effect of drought stress and cultivar (Table 1). The effect of drought stress was greater on YL10, which had shorter panicles with lower dry mass than HQR (Supplementary Figure S2). Drought stress stimulated significantly the diameter of the culm of HQR compared with that of the control, whereas the diameter of the culm of YL10 was not (Supplementary Table S2).

**Table 1:** Results of two-way ANOVA on the effects of cultivar and drought stress on agronomic traits of broomcorn millet as dependent variables (*n* = 3).

	Cultivar		Drought stress		Cultivar × Drought stress	
	*F*	*p* value	*F*	*p* value	*F*	*p* value
Plant height (cm)	0.104	0.755	22.435	0.001	0.739	0.415
Number of internode	20.167	0.002	0.167	0.694	1.500	0.256
Culm diameter (cm)	74.064	<0.001	23.170	0.001	0.191	0.673
Panicle length (cm)	4.009	0.080	51.136	<0.001	21.827	0.002
Dry weight of panicle (g)	5.516	0.047	45.991	<0.001	0.298	0.600
Grain weight per plant (g)	1.353	0.278	42.148	<0.001	0.023	0.883

### Variation in the α Diversity and Composition of Bacterial Rhizosphere Community

Across treatments, the number of OTUs varied from 1515.3 ± 52.5 to 3004.7 ± 249.9 (mean ± SD), and the Shannon index ranged from 7.1 ± 1.3 to 9.3 ± 0.4. The effects of plant developmental stage, cultivar, and drought stress on the diversity of the bacterial rhizosphere community varied (Table 2). Developmental stage had a significantly negative effect on the number of OTUs and Shannon index. However, no significant effect of cultivar, drought stress, or their interaction on either metric was observed (Table 2).

**Table 2:** Results of three-way ANOVA on the effects of cultivar, drought stress, and developmental stage on the richness and evenness of the bacterial community within the rhizosphere of broomcorn millet (*n* = 3).

Factor	OTU number		Shannon index	
	*F*	*p* value	*F*	*p* value
Cultivar	1.235	0.277	2.854	0.104
Developmental stage	30.647	<0.001	3.969	0.032
Drought	0.203	0.657	0.021	0.887
Cultivar × Drought	2.243	0.147	1.244	0.276
Cultivar × Developmental stage	0.050	0.951	0.126	0.882
Developmental stage × Drought	1.588	0.225	1.103	0.348
Cultivar × Developmental stage × Drought	2.410	0.111	1.358	0.276

**FIGURE 1:**
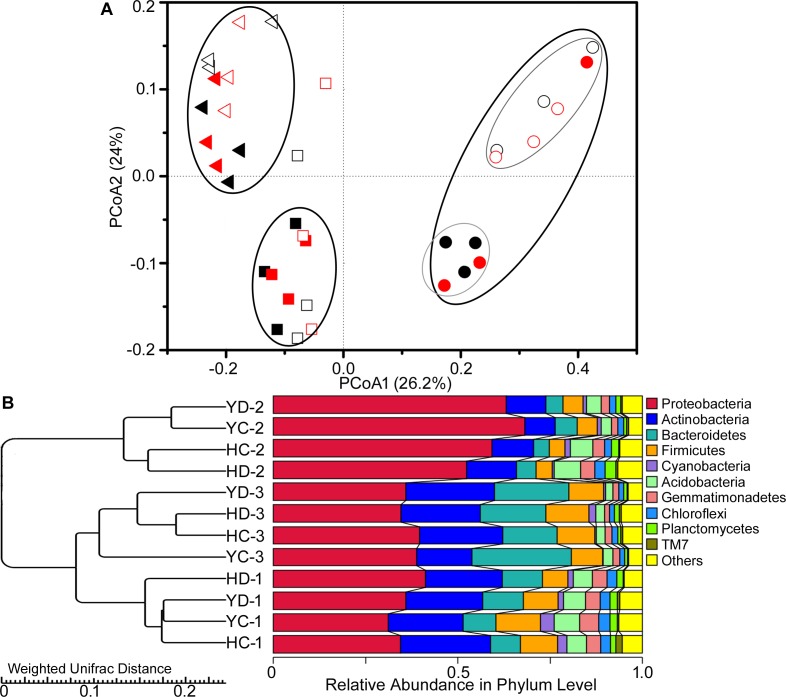
Variations in the composition of the bacterial rhizosphere community of HQR and YL10 at distinct developmental stages and under drought stress. **(A)** Principal coordination analysis (PCoA) based on the weighted UniFrac distances. Square, jointing stage; circular, flowering stage; triangle, grain filling stage. Black, control; red, drought treatment. The solid square, circular, and triangle represent HQR, whereas the open ones represent YL10. **(B)** UPGMA clustering using Weighted UniFrac Distances. H = HQR; Y = YL10; C = control, D = drought stress; 1 = jointing stage; 2 = flowering stage; 3 = grain filling stage.

Similarly, the rhizosphere bacterial community showed variation in the PCoA community structure as a function of plant developmental stage (Figure 1A). At the development stages, the result of variance partitioning demonstrated that 12.3% of variance could be explained by the developmental stage (adjusted *R*^2^ = 12.3%, *F* = 5.6, *p* = 0.004), whereas the effect of drought (adjusted *R*^2^ = 0.0%, *F* = 0.6, *p* = 0.682) or cultivar (adjusted *R*^2^ = 0.8%, *F* = 1.3, *p* = 0.232) was not significant. At the flowering stage, the rhizobacterial assemblages of HQR and YL10 were obviously different from each other (Figure 1A). Cultivar explained a small but significant fraction of the total variation in the bacterial rhizosphere community composition at the specific stage (adjusted *R*^2^ = 8.0%, *F* = 1.9, *p* = 0.036). UPGMA analysis showed the same trends as that of PCoA (Figure 1B). Rhizobacterial communities from HQR and YL10 rhizospheres at the jointing and grain filling stages were clustered into one clade. The bacterial community structures of these samples were more similar to one another compared with that of the samples collected at the flowering stage (Figure 1B).

### Variation in Bacterial Abundance

We recorded a total of 923 core OTUs in all of the samples, which were composed of Proteobacteria (33.8%), Actinobacteria (21.0%), Acidobacteria (10.7%), Bacteroidetes (8.2%), Chloroflexi (6.3%), Gemmatimonadetes (5.9%), Firmicutes (3.5%), Verrucomicrobia (2.9%), and Planctomycetes (2.7%, Supplementary Figure S3). Across all samples, these nine bacterial phyla, together with TM7, were the 10 dominant phyla detected in this study, which represented 93.6–96.3% of the assigned sequences (Supplementary Table S3). The relative abundances of these bacterial phyla in each rhizosphere varied with plant developmental stage except Cyanobacteria and TM7, which were consistently present at 1.31 and 0.55%, respectively (Supplementary Table S3).

**FIGURE 2:**
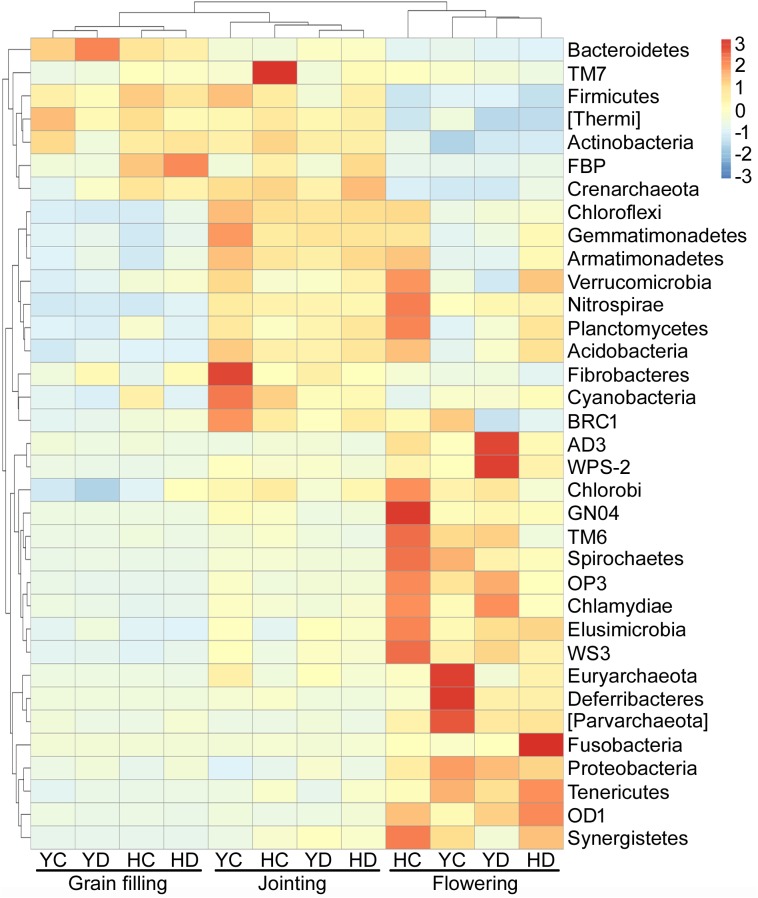
Distribution heat map of bacterial phyla arranged in terms of the developmental stage of broomcorn millet. H = HQR; Y = YL10; C = control, D = drought stress.

The variations in the relative abundances of bacterial phyla were further analyzed because the developmental stage of broomcorn millet drove the changes in the community composition of rhizosphere bacteria (Figure 1), and three distinct groups of bacteria with respect to developmental stage were found (Figure 2). The relative abundances of Proteobacteria, AD3, WPS-2, Chlorobi, GN04, TM6, Spirochaetes, OP3, Chlamydiae, Elusimicrobia, WS3, Euryarchaeota, Deferribacteres, Parvarchaeota, Fusobacteria, Tenericutes, OD1, and Synergistetes were enriched at the flowering stage relative to the jointing and grain filling stages (Figure 2). Actinobacteria, Bacteroidetes, Firmicutes, and Thermi were enriched in the jointing and grain filling stages (Figure 2). The third group, including Acidobacteria, Chloroflexi, Gemmatimonadetes, Planctomycetes, Cyanobacteria, Verrucomicrobia, Nitrospirae, Armatimonadetes, Fibrobacteres, and BRC1, were specifically enriched at the jointing stage and then decreased with plant development (Figure 2).

**FIGURE 3:**
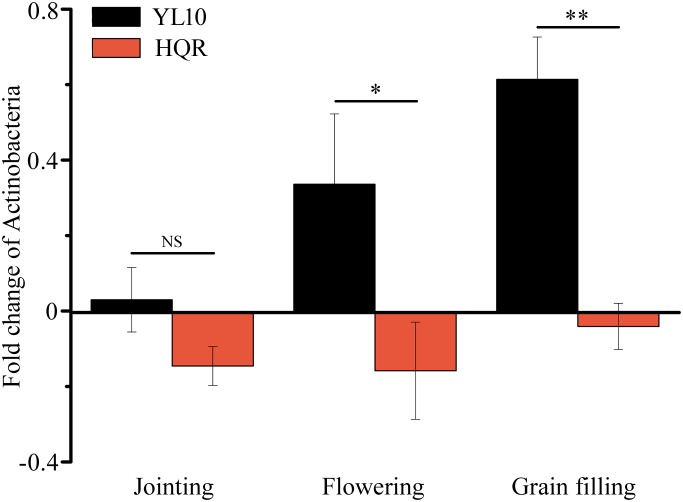
Effect of drought stress on the relative abundance of Actinobacteria in the rhizosphere of broomcorn millet. The fold change of Actinobacteria was calculated using the following equation: (relative abundance under drought/relative abundance under control) –1. Error bars are standard error over three independent replicates (*n* = 3). NS indicates no significant difference between HQR and YL10; ^∗^ *p* < 0.05; ^∗∗^ *p* < 0.01.

**FIGURE 4:**
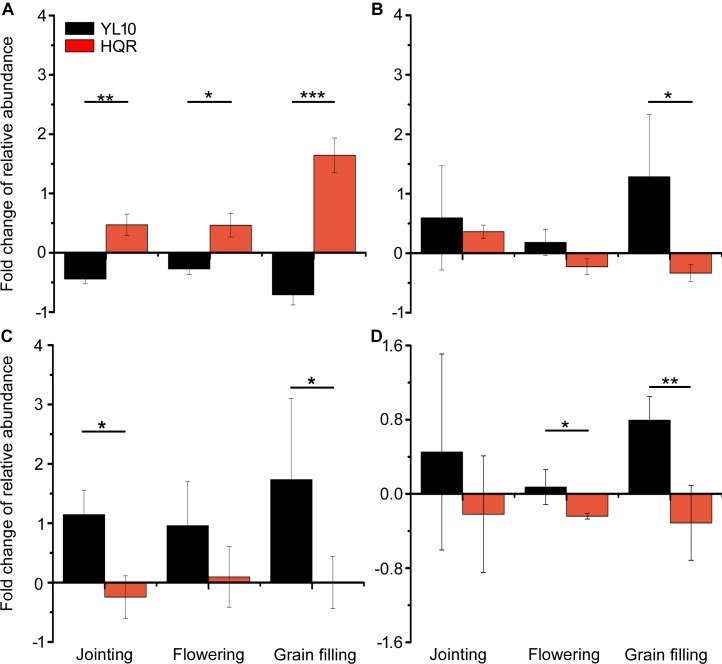
Effects of drought stress on the relative abundances of *Acinetobacter* **(A)**, *Lysobacter* **(B)**, *Streptomyces* **(C)**, and *Cellvibrio* **(D)** in the rhizosphere of broomcorn millet. The fold change of each bacterial genera was calculated using the following equation: (relative abundance under drought/relative abundance under control) –1. Error bars are standard error of three independent replicates (*n* = 3). ^∗^*p* < 0.05; ^∗∗^*p* < 0.01; ^∗∗∗^*p* < 0.001.

A significant effect of cultivar on the response of Actinobacteria to drought stress was detected through two-way ANOVA (Supplementary Table S4). Actinobacteria mainly accumulated in the rhizosphere of YL10, whereas an opposite pattern was found in the rhizosphere of HQR under drought stress (Figure 3). Five of the 50 most dominant bacterial genera were affected by the cultivar and/or developmental stage of broomcorn millet (Supplementary Table S5). In these genera, developmental stage significantly affected the responses of *Adhaeribacter* and *Acinetobacter* to drought stress, whereas cultivar influenced the responses of *Lysobacter*, *Cellvibrio*, *Streptomyces*, and *Acinetobacter* (Supplementary Table S5). The ratio of drought-exposed *Acinetobacter* to the control was also significantly affected by the interactive effect of developmental stage and cultivar (Supplementary Table S5). The relative abundance of *Acinetobacter* was notably upregulated in the rhizosphere of HQR under drought stress but was downregulated in the rhizosphere of YL10 (Figure 4A). By contrast, the relative abundances of *Lysobacter*, *Streptomyces*, and *Cellvibrio* in the rhizosphere of YL10 were mainly stimulated by drought stress compared with that of HQR (Figure 4B–D).

## Discussion

### Plant Stage Is the Major Factor Driving the Succession of Bacterial Rhizosphere Community of Broomcorn Millet

The effects of plant developmental stage on the diversity, dynamics, and composition of bacterial rhizosphere communities have been comprehensively elucidated. For example, the growth of a seedling to a mature plant significantly affects the microbial community structure in the rhizosphere of various plants, including *Arabidopsis* (Micallef et al., 2009; Chaparro et al., 2014), maize (Baudoin et al., 2003; Haichar et al., 2008), sweet potato (Marques et al., 2014), pea, wheat, sugar beet (Houlden et al., 2008), and *Caragana liouana* (Na et al., 2017a). In our study, similar effects were observed, and significant changes in the rhizosphere bacterial community structure and α diversity were found as the millet matured from the seedling to the grain filling stage (Figure 1 and Table 2).

Changes in bacterial communities may be explained by the outputs of the plants themselves. Root exudate quantity, quality, and exudation rate vary with plant development and rapidly affect the growth of specific microorganisms in the rhizosphere, especially fast-growing microbes, and can cause structural divergences (Aulakh et al., 2001; Baudoin et al., 2003; Chaparro et al., 2014). As such, the effects of plant developmental stages on microbial community diversity and composition may be mediated by the varying root exudation patterns (Dunfield and Germida, 2003; Chaparro et al., 2014).

As broomcorn millet matured, the richness and evenness of the bacterial community in the rhizosphere gradually decreased. These results could be attributed to the change in the relative allocation of photosynthesis products between above and belowground parts of the plants (Brady and Weil, 1999; Brimecombe et al., 2001). After the flowering stage, the photosynthates are mainly used to maintain seed development and less are allocated to root exudates (Brady and Weil, 1999; Lucas García et al., 2010). This variation in root exudates may then disrupt the recruitment of bacteria and weaken the dominance of copiotrophic populations in the rhizosphere (Figure 2).

The changes in root exudation patterns might drive specific rhizosphere bacterial assemblages based on the developmental stage of the millet. The unique bacterial rhizosphere assemblage of each stage might represent the specific physiological needs or requirements of broomcorn millet at a particular stage because root exudates and physiological status varied with plant development (Brady and Weil, 1999; Brimecombe et al., 2001), driving the colonization of particular functional microbial populations in the rhizosphere (Wasson et al., 2006). For example, the enrichment of some less dominant bacteria at the flowering stage relative to the jointing and grain filling stages indicated a specific variation in the diversity and quantity of root depositions during plant flowering (Figure 3). Plants can secrete high levels of low-molecular-weight organic acids to the rhizosphere to acquire more nutrients during flowering than fruiting (Aulakh et al., 2001; Lucas García et al., 2010). Aulakh et al. (2001) also suggested that the highest exudation rates are detected at the flowering stage among the stages of the life cycle of rice.

### Stage-Dependent Effects of Cultivar on the Development of Bacterial Rhizosphere Community Composition

Comparisons between plant species and cultivars have demonstrated the differences in microbial rhizosphere communities when community structure and function are considered at specific times (Haichar et al., 2008; Peiffer et al., 2013; Marques et al., 2014). In the present study, the PCoA results showed obvious differences in the structure of the bacterial rhizosphere community between HQR and YL10 at the flowering stage only (Figure 1A). This result demonstrated that the effects of cultivars on the rhizobacterial community composition of broomcorn millet were plant stage dependent. Previous studies confirmed that root exudation pattern (i.e., in both quantity and quality) among species is genetically controlled (Rengel, 2002). As such, this unique cultivar effect of broomcorn millet on its rhizosphere bacterial community composition might precisely reflect the genetic difference in HQR and YL10. From this perspective, the most obvious divergence in physiological status during the life cycle of HQR and YL10 might occur at the flowering stage, following the results of the PCoA.

However, we have yet to determine why the variation in community structure occurs only at the flowering stage of the two cultivars. The flowering stage is the transition phase from vegetative growth to reproductive growth in herbaceous plants. After the flowering stage, photosynthesis products are mainly allocated to and accumulated in the seeds (Aulakh et al., 2001). Our results showed that the plant height and culm diameter of HQR were larger than those of YL10, whereas the panicle length of HQR was significant shorter than that of YL10; nevertheless, they had the same grain weight per plant (Supplementary Table S2). These results indicated that YL10 allocated more carbon to increase grain yield relative to that of HQR after flowering. As such, the differences in photosynthate redistribution between the two broomcorn millet cultivars at the flowering stage could partly explain the divergence of bacterial rhizosphere community composition. Another reason might be the differences in the concentration and composition of the root exudates of the two cultivars (Aulakh et al., 2001; Lucas García et al., 2010).

### Bacterial Rhizosphere Community of Broomcorn Millet Is Robust Under Drought Stress

The results of a commonly used drought-stress method (Earl, 2003) showed that neither the α diversity nor the composition of the bacterial rhizosphere community of the two broomcorn millet cultivars changed in response to drought stress (Figure 1 and Table 2). These results were inconsistent with our hypothesis 2, as well as the results of previous studies, suggesting that soil moisture is an important factor that affects the composition of soil microbial communities (Griffiths et al., 2011; Na et al., 2017b). Three reasons might explain this paradox. First, 15 days of drought treatment might be too short to affect the rhizosphere bacterial community structure of broomcorn millet. Although the growth of broomcorn millet was significantly inhibited under drought stress (Supplementary Table S2), the rhizosphere bacterial communities did not respond to the lack of water. Second, broomcorn millet only requires a small amount of water for growth and development (Baltensperger, 2002; Lágler et al., 2005; Jana and Jan, 2006; Gulati et al., 2016). As such, a short period of drought stress at the seedling stage might not affect the interactions between the root of broomcorn millet and the associated soil microbes in the rhizosphere. Third, the diversity and structure of rhizobacterial community detected under control and drought stress conditions might represent the normal status of broomcorn millet grown in arid and semi-arid areas. Thus, the bacterial rhizosphere community of broomcorn millet as a whole was robust under drought. The drought tolerance of broomcorn millet and its local microbiota might be indicative of co-evolutionary mechanisms that have occurred over time.

### Cultivar-Dependent Responses of Specific Rhizosphere Bacterial Populations to Drought Stress

Xu et al. (2018) demonstrated that drought stress stimulates the abundance and activity of monoderm bacteria, such as Actinobacteria and Firmicutes, in the rhizosphere of *Sorghum bicolor* via the shifts in root metabolism. Consistent with this finding, our results confirmed that a proportion of Actinobacteria accumulated in the rhizosphere of YL10 under drought stress (Figure 3). However, the response pattern of Actinobacteria differed between HQR and YL10 (Figure 3), which inferred that the recruiting mechanism of these bacterial species depended on the cultivar or genotype of host plants. Xu et al. (2018) suggested that this difference may be associated with the altered metabolism of roots under drought. Further metabolic and genetic experimentations would help verify this hypothesis.

Actinobacteria species have great economic importance to humans because of their functions in agricultural and forest soil ecosystems, such as organic matter decomposition (Steger et al., 2007), nitrogen fixation (Gtari et al., 2007), antibiotic production (Jiang et al., 2017), and plant defense induction (Conn et al., 2008). *Streptomyces* and *Lysobacter* are widely studied bacterial genera in the production of a wide range of antibiotic and bioactive secondary metabolites that may be useful against phytopathogens (Haruo et al., 2003; Hashizume et al., 2004). *Acinetobacter* spp. exhibit plant growth-promoting properties, including phosphate solubilization, nitrogen fixation, and auxin production (Gulati et al., 2009; Sachdev et al., 2010). Consistent with the functions of these bacteria, our results indicated that HQR might maintain its growth performance under drought stress by recruiting plant growth-promoting bacteria, whereas YL10 might recruit antibiotic-producing bacterial species. HQR strengthened its ability to acquire nutrients, whereas YL10 enhanced its health status in the rhizosphere under drought stress. Although this observation was not confirmed by our study, our results suggested that the two cultivars of broomcorn millet might have evolved distinct response mechanisms to drought stress through interactions with specific bacterial populations. The influence of plant–microbe interactions in the rhizosphere on the drought tolerance of broomcorn millet, as well as the underlying genetic mechanism controlling these processes, should be further examined.

Given that cultivar and drought stress had no significant effect on the α diversity and composition of bacterial rhizosphere community (Figure 1 and Table 2), considering the functions of specific microbes in the rhizosphere might be more important than comparing the bacterial rhizosphere community as a whole to understand the drought resistance mechanism of broomcorn millet. Further metabolomic, metagenomic, and metatranscriptomic analyses are needed to assess the function of microbial associations to understand the co-evolution mechanisms between this oldest drought-tolerant crop and its local microbiota.

## Conclusion

With Illumina MiSeq technology, our study revealed that (1) plant stage was the major driver of the α diversity and structure of bacterial rhizosphere communities of two broomcorn millet cultivars; (2) the effect of broomcorn millet cultivar on the bacterial community composition was stage dependent; and (3) the bacterial rhizosphere community of both cultivars was robust to drought stress. Overall, these results might contribute to our understanding of the co-evolutionary mechanisms of drought-tolerant broomcorn millet and its associated microbiome.

## Author Contributions

XN and XC designed the research. XC, SL, JW, HW, and LC carried out the pot experiments. XN, CM, SM, and PX performed DNA extraction and detection. XN analyzed the data and drafted the manuscript. XC and ZQ supervised the study and participated in its coordination. All authors read and approved the final manuscript.

## Conflict of Interest Statement

The authors declare that the research was conducted in the absence of any commercial or financial relationships that could be construed as a potential conflict of interest.

## Footnotes

1 http://drive5.com/uparse/
