# Supplementary material for: Plant Stage, Not Drought Stress, Determines the Effect of Cultivars on Bacterial Community Diversity in the Rhizosphere of Broomcorn Millet (Panicum miliaceum L.)
Source: Front Microbiol. 2019 Apr 24;10:828. doi: 10.3389/fmicb.2019.00828 (PMC6491785; doi:10.3389/fmicb.2019.00828)
Supplement: Supplementary file 1 [file Table_1.doc]

Supplementary Materials

**Plant age, but not drought stress, determined the effects of cultivar on bacterial community dynamics in the rhizosphere of broomcorn millet (*Panicum miliaceum* L.)**

**Xiaofan Na^1§*^, Xiaoning Cao^2,3§*^, Caixia Ma^1^, Shaolan Ma^1^, Pengxin Xu^1^, Sichen Liu^2,3^, Junjie Wang^2,3^, Haigang Wang^2,3^, Ling Chen^2,3^, Zhijun Qiao^2,3^**

*** Correspondence:** Xiaofan Na: [nxf-0324@163.com](mailto:nxf-0324@163.com); Xiaoning Cao: [sxnkypzscxn@sina.com](mailto:sxnkypzscxn@sina.com)


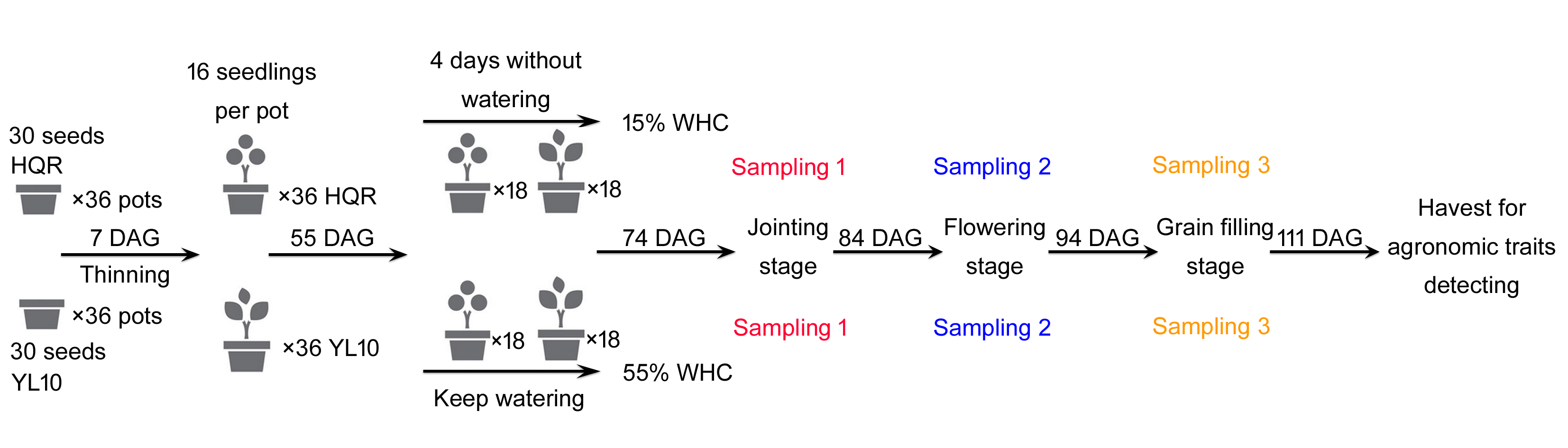


**Supplementary Figure S1** The timeline scheme of the sampling procedures in this study. HQR indicates the broomcorn millet cultivar *HeQu Red*; YL10, the broomcorn millet cultivar *YanLi 10*; DAG, days after germination; WHC, water-holding capacity.


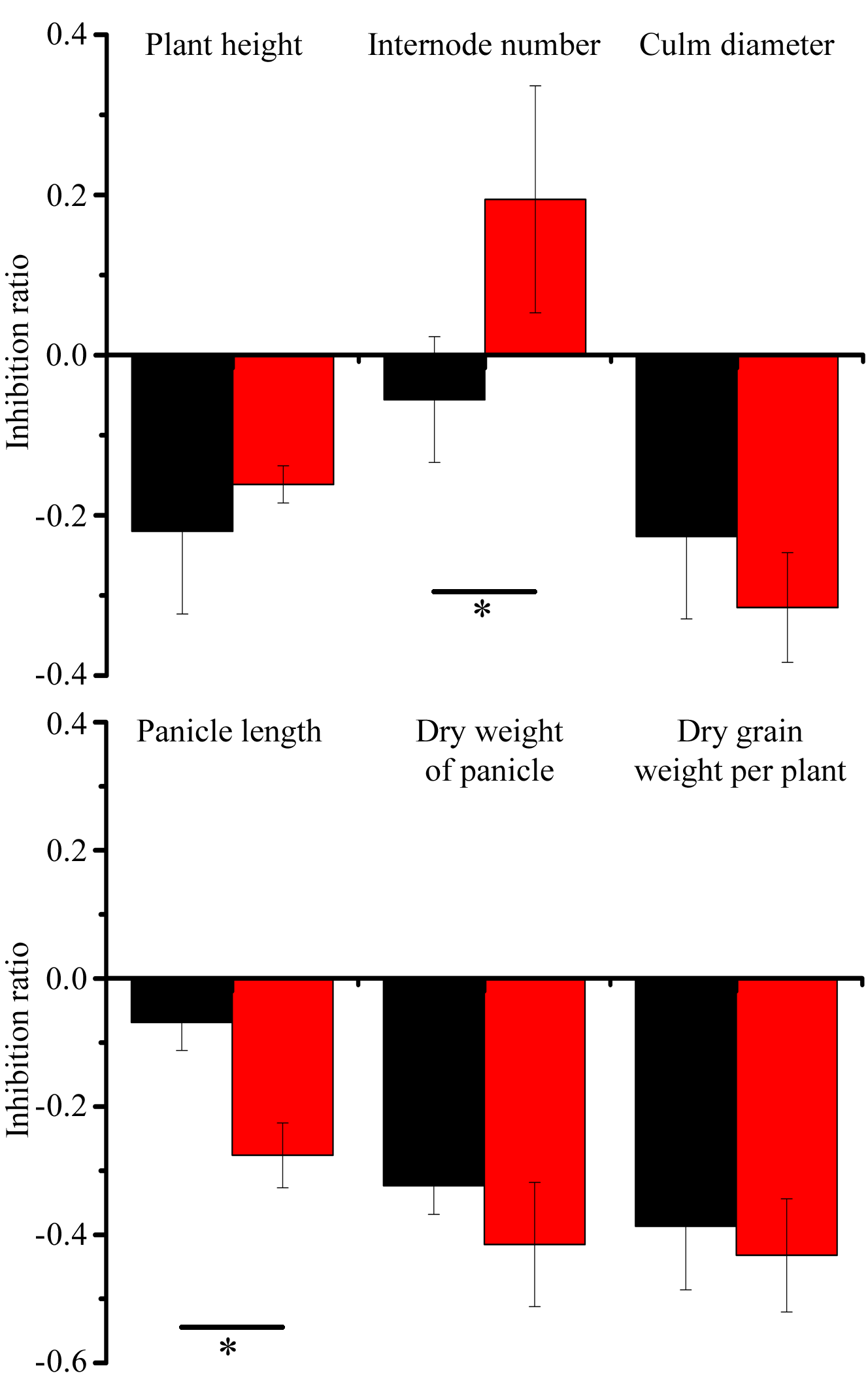


**Supplementary Figure S2** The effects of drought treatment on the major agronomic traits of broomcorn millet. The inhibition ratio was calculated by (data under drought/data under control) – 1. Black columns represent the cultivar HQR, red columns represent YL10. Error bars are standard error over three independent replicates. * *p* < 0.05; *t* test.

**
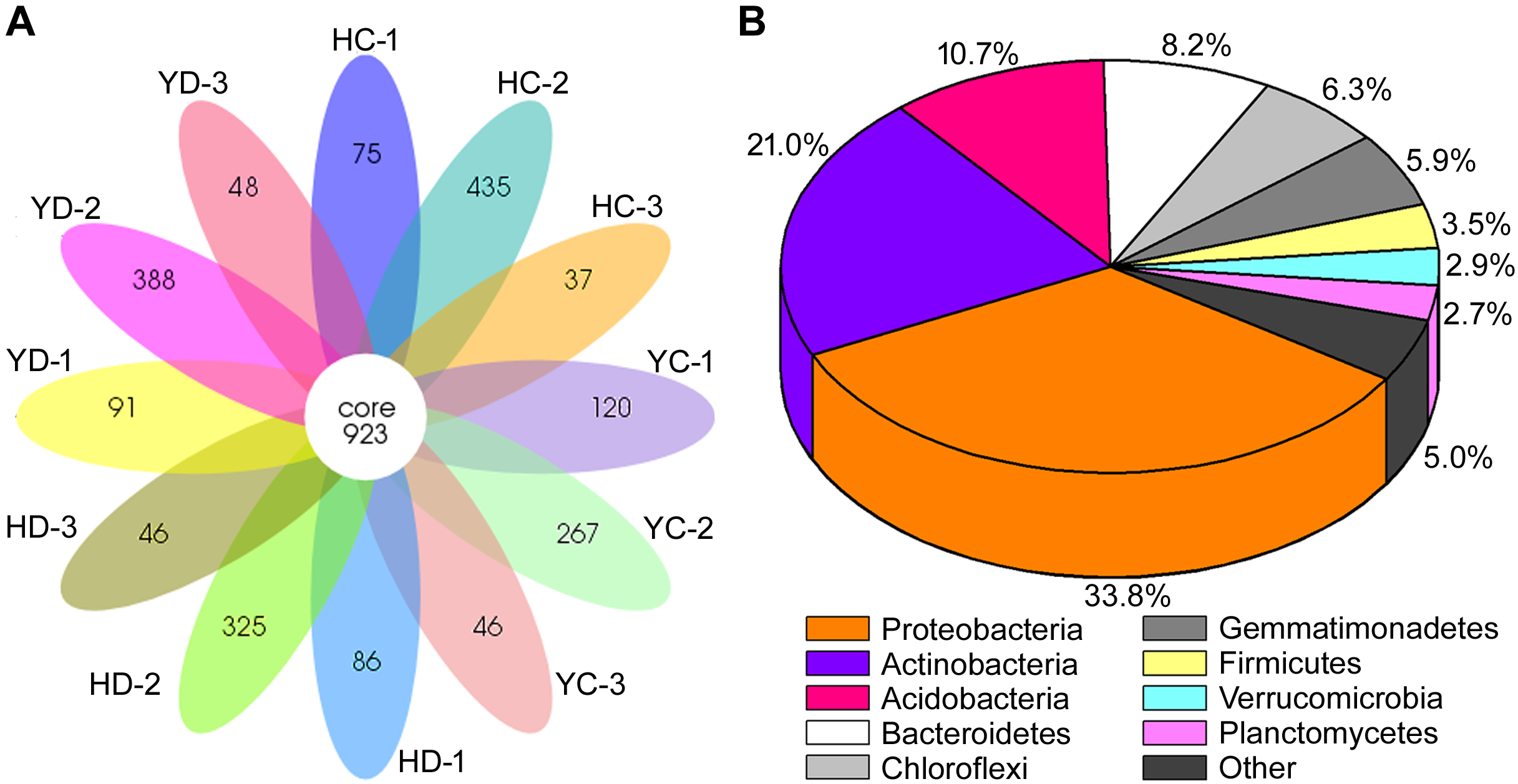
**

**Supplementary Figure S3** The composition of core OTUs in rhizosphere of broomcorn millet. (A) Flower diagram of the OTUs detected in rhizosphere soils of bacterial communities sampled from different development stages, cultivars and under drought conditions of broomcorn millet. H represents *HeQu Red*; Y, *YanLi 10*; C, control, D, drought stress; 1, jointing stage; 2, flowering stage; 3, grain filling stage. (B) The proportions of the core OTUs in rhizosphere of broomcorn millet at phyla level.

**Table S1** Statistics of quality control of the Barcoded Illumina MiSeq 2500 sequencing results.

|  | HRQ | | | | | | YL10 | | | | | |
| --- | --- | --- | --- | --- | --- | --- | --- | --- | --- | --- | --- | --- |
|  | Jointing | | Flowering | | Grain filling | | Jointing | | Flowering | | Grain filling | |
|  | Control | Drought | Control | Drought | Control | Drought | Control | Drought | Control | Drought | Control | Drought |
| Mean raw reads | 66742 | 58926 | 58638 | 50864 | 58884 | 62155 | 59817 | 63081 | 47615 | 53566 | 61017 | 65096 |
| Mean high quality reads | 64524 | 57027 | 43578 | 41955 | 57268 | 60465 | 57847 | 60894 | 38638 | 36764 | 59204 | 63081 |
| Mean taxon tags | 52791 | 45889 | 37034 | 35783 | 43977 | 46549 | 49035 | 50632 | 33239 | 30133 | 50708 | 47768 |
| Mean read length | 253 | 253 | 255 | 255 | 253 | 253 | 254 | 254 | 255 | 246 | 253 | 253 |
| Mean GC content (%) | 55.7 | 55.4 | 55.8 | 55.9 | 54.9 | 54.7 | 55.8 | 55.5 | 55.8 | 56.0 | 53.9 | 54.8 |
| Mean OTU number | 3653 | 3575 | 3717 | 3087 | 2206 | 2185 | 3732 | 3697 | 2654 | 2899 | 2305 | 2260 |
| Mean effective (%) | 87.4 | 86.9 | 70.6 | 75.9 | 83.6 | 83.7 | 89.2 | 87.6 | 74.0 | 62.0 | 89.2 | 81.8 |

**Table S2** Major agronomic traits of broomcorn millet cultivars HRQ and YL10 under control and drought stress. Data shown are means ± SD (*n* = 3). Different letters indicate significant differences (*p* < 0.05) as determined by Tukey HSD test.

|  | HQR | | YL10 | |
| --- | --- | --- | --- | --- |
|  | Control | Drought | Control | Drought |
| Plant height (cm) | 91.2 ± 9.3a | 70.3 ± 4.4b | 89.2 ± 6.0a | 74.7 ± 5.0ab |
| Number of internode | 5.7 ± 0.6a | 5.3 ± 0.6a | 3.3 ± 0.6b | 4.0 ± 1.0ab |
| Culm diameter (cm) | 0.4 ± 0.05b | 0.5 ± 0.05a | 0.2 ± 0.01ac | 0.3 ± 0.02c |
| Panicle length (cm) | 31.5 ± 1.3b | 29.3 ± 1.9bc | 37.3 ± 0.8a | 27.0 ± 1.8c |
| Dry weight of panicle (g) | 3.3 ± 0.2a | 2.2 ± 0.1bc | 3.0 ± 0.5ab | 1.7 ± 0.3c |
| Dry grain weight per plant (g) | 2.5 ± 0.2a | 1.5 ± 0.2b | 2.4 ± 0.4a | 1.3 ± 0.2b |

**Table S3** The mean relative abundance (± SD; *n* = 3) of the dominant rhizosphere bacterial phyla of HRQ and YL10 at different development stages*.* Different letters indicate significant differences (*p* < 0.05) as determined by Tukey HSD test.

| Taxonomy | HQR | | | | | | YL10 | | | | | |
| --- | --- | --- | --- | --- | --- | --- | --- | --- | --- | --- | --- | --- |
|  | Jointing | | Flowering | | Grain filling | | Jointing | | Flowering | | Grain filling | |
|  | Control | Drought | Control | Drought | Control | Drought | Control | Drought | Control | Drought | Control | Drought |
| Proteobacteria | 34.5 ± 1.5b | 35.9 ± 2.7b | 52.3 ± 2.2ab | 59.3 ± 13.8ac | 39.6 ± 8.8b | 34.6 ± 2.4b | 31.1 ± 6.6b | 41.2 ± 6.8bc | 68.1 ± 10.8a | 63.0 ± 7.3a | 38.9 ± 9.1b | 35.9 ± 1.5b |
| Actinobacteria | 24.4 ± 0.6a | 20.8 ± 3.3abc | 13.5 ± 1.6bcd | 11.2 ± 5.1cd | 22.5 ± 0.8ab | 21.5 ± 3.8ab | 20.3 ± 0.5abc | 20.8 ± 4.7abc | 8.3 ± 2.4d | 10.8 ± 5.0cd | 14.9 ± 1.8acd | 24.0 ± 5.6a |
| Bacteroidetes | 8.1 ± 1.5cd | 11.1 ± 3.2bcd | 5.3 ± 3.2d | 4.3 ± 0.2d | 14.8 ± 3.5bc | 17.7 ± 0.9b | 8.9 ± 1.2cd | 10.8 ± 4.1bcd | 6.0 ± 2.4d | 4.8 ± 0.9d | 27.0 ± 2.6a | 20.3 ± 6.3ab |
| Firmicutes | 10.0 ± 6.6 | 9.4 ± 5.6 | 4.5 ± 0.5 | 4.2 ± 0.5 | 10.2 ± 6.6 | 11.7 ± 2.9 | 12.1 ± 10.7 | 7.0 ± 1.9 | 5.4 ± 2.6 | 5.3 ± 1.1 | 8.5 ± 5.4 | 9.5 ± 2.8 |
| Cyanobacteria | 2.6 ± 0.9 | 1.5 ± 0.9 | 0.5 ± 0.2 | 1.4 ± 1.1 | 0.3 ± 0.1 | 1.8 ± 1.3 | 3.6 ± 5.4 | 1.4 ± 1.3 | 1.1 ± 0.4 | 1.0 ± 0.3 | 0.2 ± 0.1 | 0.4 ± 0.2 |
| Acidobacteria | 5.4 ± 1.3 | 5.9 ± 0.7 | 7.2 ± 1.8 | 6.1 ± 3.3 | 2.4 ± 0.4 | 2.3 ± 0.3 | 6.9 ± 3.0 | 5.2 ± 3.3 | 2.7 ± 0.6 | 4.0 ± 0.7 | 2.6 ± 0.3 | 2.0 ± 0.8 |
| Gemmatimonadetes | 3.8 ± 1.1ab | 3.9 ± 0.8ab | 3.9 ± 0.7ab | 3.2 ± 1.6ab | 1.9 ± 0.7b | 1.4 ± 0.1b | 5.3 ± 2.1a | 4.0 ± 2.1ab | 1.7 ± 0.3b | 2.2 ± 0.3ab | 1.9 ± 0.1b | 1.6 ± 0.5b |
| Chloroflexi | 2.7 ± 0.4 | 2.8 ± 0.6 | 2.8 ± 0.6 | 1.9 ± 0.6 | 1.6 ± 0.4 | 1.3 ± 0.2 | 3.0 ± 1.2 | 2.7 ± 1.3 | 1.6 ± 0.3 | 1.8 ± 0.1 | 1.3 ± 0.2 | 1.4 ± 0.3 |
| Planctomycetes | 1.4 ± 0.4ab | 2.0 ± 0.3ab | 2.9 ± 0.9a | 2.0 ± 0.8ab | 0.9 ± 0.2b | 1.3 ± 0.6ab | 1.9 ± 0.8ab | 1.7 ± 0.9ab | 0.9 ± 0.4b | 1.3 ± 0.5ab | 0.8 ± 0.3b | 0.8 ± 0.2b |
| TM7 | 1.8 ± 1.7 | 0.6 ± 0.2 | 0.5 ± 0.3 | 0.3 ± 0.1 | 0.5 ± 0.3 | 0.6 ± 0.4 | 0.4 ± 0.2 | 0.4 ± 0.2 | 0.5 ± 0.3 | 0.4 ± 0.1 | 0.3 ± 0.2 | 0.3 ± 0.2 |
| Others | 5.4 ± 2.0 | 6.1 ± 2.2 | 6.7 ± 1.3 | 6.1 ± 2.7 | 5.3 ± 2.7 | 5.8 ± 1.1 | 6.4 ± 2.9 | 4.9 ± 3.4 | 3.7 ± 1.6 | 5.5 ± 4.0 | 3.7 ± 1.8 | 3.9 ± 2.9 |

**Table S4** Results of a two-way ANOVA on the effects of cultivar and developmental stage on the responses of dominant bacterial phyla to drought stress.

| Phyla | DS | | | Cultivar | | | DS × Cultivar | | |
| --- | --- | --- | --- | --- | --- | --- | --- | --- | --- |
|  | *df* | *F* | *p* value | *df* | *F* | *p* value | *df* | *F* | *p* value |
| Proteobacteria | 2 | 1.771 | 0.212 | 1 | 0.292 | 0.599 | 2 | 1.574 | 0.247 |
| Actinobacteria | 2 | 1.538 | 0.254 | 1 | 7.536 | **0.018** | 2 | 0.766 | 0.486 |
| Bacteroidetes | 2 | 1.110 | 0.361 | 1 | 1.017 | 0.333 | 2 | 0.255 | 0.779 |
| Firmicutes | 2 | 0.350 | 0.711 | 1 | 0.132 | 0.723 | 2 | 0.388 | 0.686 |
| Cyanobacteria | 2 | 2.210 | 0.152 | 1 | 1.579 | 0.233 | 2 | 0.767 | 0.486 |
| Acidobacteria | 2 | 0.771 | 0.484 | 1 | 0.023 | 0.883 | 2 | 1.598 | 0.242 |
| Gemmatimonadetes | 2 | 0.491 | 0.624 | 1 | 0.216 | 0.651 | 2 | 1.115 | 0.360 |
| Chloroflexi | 2 | 0.173 | 0.843 | 1 | 1.372 | 0.264 | 2 | 0.608 | 0.560 |
| Planctomycetes | 2 | 0.041 | 0.960 | 1 | 0.013 | 0.912 | 2 | 1.672 | 0.229 |
| TM7 | 2 | 0.065 | 0.938 | 1 | 0.214 | 0.652 | 2 | .901 | 0.432 |

DS indicates developmental stage; Bold case represents the significance at *p* < 0.05 level.

**Table S5** Results of a two-way ANOVA on the effects of cultivar and developmental stage on the responses of dominant bacterial genera to drought stress.

| Bacterial genera | Developmental stage | | | Cultivar | | | Developmental stage×Cultivar | | |
| --- | --- | --- | --- | --- | --- | --- | --- | --- | --- |
|  | *df* | *F* | *p* | *df* | *F* | *p* | *df* | *F* | *p* |
| *Halomonas* | 2 | 0.782 | 0.479 | 1 | 0.264 | 0.616 | 2 | 1.266 | 0.317 |
| *Arthrobacter* | 2 | 1.244 | 0.323 | 1 | 3.317 | 0.094 | 2 | 0.014 | 0.987 |
| *Pseudomonas* | 2 | 0.947 | 0.415 | 1 | 2.150 | 0.168 | 2 | 0.752 | 0.492 |
| *Shewanella* | 2 | 0.755 | 0.493 | 1 | 1.015 | 0.335 | 2 | 0.869 | 0.446 |
| *Pontibacter* | 2 | 1.510 | 0.260 | 1 | 1.005 | 0.336 | 2 | 0.409 | 0.673 |
| *Kaistobacter* | 2 | 1.567 | 0.249 | 1 | 0.679 | 0.426 | 2 | 0.046 | 0.955 |
| *Sphingobacterium* | 2 | 0.461 | 0.642 | 1 | 1.404 | 0.259 | 2 | 0.609 | 0.560 |
| *Sphingobium* | 2 | 0.465 | 0.639 | 1 | 0.097 | 0.760 | 2 | 1.313 | 0.305 |
| *Lysobacter* | 2 | 1.017 | 0.391 | 1 | **5.197** | **0.042** | 2 | 1.732 | 0.218 |
| *Gillisia* | 2 | 0.329 | 0.726 | 1 | 1.261 | 0.284 | 2 | 0.088 | 0.917 |
| *Steroidobacter* | 2 | 1.159 | 0.347 | 1 | 0.136 | 0.719 | 2 | 0.464 | 0.640 |
| *Nitrososphaera* | 2 | 0.588 | 0.572 | 1 | 1.633 | 0.288 | 2 | 0.683 | 0.525 |
| *Flavisolibacter* | 2 | 0.689 | 0.516 | 1 | 1.959 | 0.187 | 2 | 0.223 | 0.803 |
| *Sphingomonas* | 2 | 0.462 | 0.641 | 1 | 0.054 | 0.820 | 2 | 0.108 | 0.898 |
| *Adhaeribacter* | 2 | **4.559** | **0.034** | 1 | 1.752 | 0.210 | 2 | 0.445 | 0.651 |
| *Rhodococcus* | 2 | 0.516 | 0.610 | 1 | 3.254 | 0.096 | 2 | 0.508 | 0.614 |
| *Paracoccus* | 2 | 1.157 | 0.347 | 1 | 0.025 | 0.877 | 2 | 0.308 | 0.741 |
| *Lactobacillus* | 2 | 0.517 | 0.609 | 1 | 1.814 | 0.203 | 2 | 0.620 | 0.554 |
| *Bacillus* | 2 | 1.029 | 0.387 | 1 | 0.746 | 0.405 | 2 | 0.112 | 0.895 |
| *Paenisporosarcina* | 2 | 3.726 | 0.055 | 1 | 2.687 | 0.127 | 2 | 1.928 | 0.188 |
| *Agrobacterium* | 2 | 0.095 | 0.910 | 1 | 0.039 | 0.848 | 2 | 0.231 | 0.797 |
| *Kribbella* | 2 | 1.379 | 0.289 | 1 | 0.428 | 0.525 | 2 | 0.294 | 0.751 |
| *Massilia* | 2 | 0.345 | 0.715 | 1 | 2.829 | 0.118 | 2 | 0.890 | 0.436 |
| *Balneimonas* | 2 | 0.411 | 0.672 | 1 | 2.828 | 0.118 | 2 | 0.369 | 0.699 |
| *DA101* | 2 | 0.230 | 0.798 | 1 | 0.388 | 0.545 | 2 | 0.378 | 0.693 |
| *Acinetobacter* | 2 | **6.935** | **0.010** | 1 | **158.522** | **< 0.001** | 2 | **23.353** | **< 0.001** |
| *Sinorhizobium* | 2 | 1.396 | 0.285 | 1 | 0.789 | 0.392 | 2 | 0.102 | 0.904 |
| *Promicromonospora* | 2 | 1.935 | 0.187 | 1 | 1.458 | 0.250 | 2 | 0.207 | 0.816 |
| *Nitrosovibrio* | 2 | 0.240 | 0.790 | 1 | 2.927 | 0.113 | 2 | 0.172 | 0.844 |
| *Mesorhizobium* | 2 | 1.230 | 0.327 | 1 | 1.095 | 0.316 | 2 | 0.054 | 0.947 |
| *Pseudoxanthomonas* | 2 | 0.548 | 0.592 | 1 | 3.162 | 0.101 | 2 | 1.299 | 0.309 |
| *Stenotrophomonas* | 2 | 0.514 | 0.611 | 1 | 0.574 | 0.463 | 2 | 0.310 | 0.739 |
| *Devosia* | 2 | 0.851 | 0.451 | 1 | 0.158 | 0.698 | 2 | 1.016 | 0.391 |
| *Pantoea* | 2 | 0.777 | 0.482 | 1 | 1.331 | 0.271 | 2 | 0.431 | 0.660 |
| *Paenibacillus* | 2 | 0.822 | 0.463 | 1 | 0.242 | 0.631 | 2 | 1.046 | 0.381 |
| *Mycobacterium* | 2 | 0.634 | 0.547 | 1 | 1.086 | 0.318 | 2 | 0.688 | 0.521 |
| *Agromyces* | 2 | 0.378 | 0.693 | 1 | 4.422 | 0.059 | 2 | 0.272 | 0.766 |
| *Rhodoplanes* | 2 | 1.766 | 0.213 | 1 | 0.373 | 0.553 | 2 | 0.957 | 0.411 |
| *Nitrospira* | 2 | 0.041 | 0.960 | 1 | 0.662 | 0.432 | 2 | 0.573 | 0.578 |
| *Pigmentiphaga* | 2 | 0.202 | 0.820 | 1 | 0.032 | 0.860 | 2 | 1.122 | 0.358 |
| *Skermanella* | 2 | 0.296 | 0.749 | 1 | 4.695 | 0.054 | 2 | 0.109 | 0.898 |
| *Rubrobacter* | 2 | 1.436 | 0.276 | 1 | 0.001 | 0.978 | 2 | 2.029 | 0.174 |
| *Chitinophaga* | 2 | 0.958 | 0.411 | 1 | 1.143 | 0.306 | 2 | 2.154 | 0.159 |
| *Streptomyces* | 2 | 0.374 | 0.695 | 1 | **10.037** | **0.008** | 2 | 0.365 | 0.701 |
| *Niastella* | 2 | 1.152 | 0.348 | 1 | 0.571 | 0.465 | 2 | 0.633 | 0.548 |
| *Novosphingobium* | 2 | 0.532 | 0.601 | 1 | 0.087 | 0.773 | 2 | 0.444 | 0.652 |
| *Virgibacillus* | 2 | 0.672 | 0.529 | 1 | 1.409 | 0.258 | 2 | 0.743 | 0.496 |
| *Achromobacter* | 2 | 0.476 | 0.638 | 1 | 1.046 | 0.336 | 2 | 0.965 | 0.355 |
| *Achromobacter* | 2 | 0.342 | 0.717 | 1 | 1.890 | 0.194 | 2 | 0.171 | 0.845 |
| *Cellvibrio* | 2 | 0.361 | 0.705 | 1 | **4.930** | **0.046** | 2 | 0.531 | 0.601 |

Bold case represents the significance at *p* < 0.05 level.
